# Improving Acetic Acid Production by Over-Expressing PQQ-ADH in *Acetobacter pasteurianus*

**DOI:** 10.3389/fmicb.2017.01713

**Published:** 2017-09-06

**Authors:** Xuefeng Wu, Hongli Yao, Lili Cao, Zhi Zheng, Xiaoju Chen, Min Zhang, Zhaojun Wei, Jieshun Cheng, Shaotong Jiang, Lijun Pan, Xingjiang Li

**Affiliations:** ^1^School of Food Science and Engineering, Hefei University of Technology Hefei, China; ^2^Key Laboratory for Agricultural Products Processing of Anhui Province, Hefei University of Technology Hefei, China; ^3^School of Chemical Engineering and Life Sciences, Chaohu University Hefei, China

**Keywords:** Pyrroquinoline quinone-dependent alcohol dehydrogenase, *Acetobacter pasteurianus* JST-S, two-dimensional gel electrophoresis, metabolic flux analysis

## Abstract

Pyrroquinoline quinone-dependent alcohol dehydrogenase (PQQ-ADH) is a key enzyme in the ethanol oxidase respiratory chain of acetic acid bacteria (AAB). To investigate the effect of PQQ-ADH on acetic acid production by *Acetobacter pasteurianus* JST-S, subunits I (adhA) and II (adhB) of PQQ-ADH were over-expressed, the fermentation parameters and the metabolic flux analysis were compared in the engineered strain and the original one. The acetic acid production was improved by the engineered strain (61.42 g L^−1^) while the residual ethanol content (4.18 g L^−1^) was decreased. Analysis of 2D maps indicated that 19 proteins were differently expressed between the two strains; of these, 17 were identified and analyzed by mass spectrometry and two-dimensional gel electrophoresis. With further investigation of metabolic flux analysis (MFA) of the pathway from ethanol and glucose, the results reveal that over-expression of PQQ-ADH is an effective way to improve the ethanol oxidation respiratory chain pathway and these can offer theoretical references for potential mechanism of metabolic regulation in AAB and researches with its acetic acid resistance.

## Introduction

Acetic acid bacteria (AAB) are employed in the industrial production of acetic acid. Their characteristics directly affect the yield and flavor of vinegar (Quintero et al., 2009). The fermentative oxidation of ethanol to acetic acid has been shown to depend on two sequential reactions of membrane-bound pyrrpquinoline quinone-dependent alcohol dehydrogenase (PQQ-ADH) and aldehyde dehydrogenase (ALDH), both of which are localized on the periplasmic side of the inner membrane (Toyama et al., 2004; Yakushi and Matsushita, 2010; Trček and Matsushita, 2013). PQQ-ADH complex in *Acetobacter pasteurianus* consist in three subunits, a dehydrogenase subunit (subunit I), a cytochrome c subunit (subunit II), the smallest one (subunit III). The subunit I encoded by adhA contains pyrrpquinoline quinone and one heme c; the subunit II encoded by adhB contains three heme c and relates to membrane-binding and ubiquinone reduction; the smallest subunit is encoded adhS, far from the operon coding for adhA and adhB, seems not to be necessary for ethanol oxidation (Kondo et al., 1995; Toyama et al., 2004). Loss of membrane-bound ADH activity resulted in reduced acetic acid resistance (Chinnawirotpisan et al., 2003). Trček et al. (2006) showed that growth characteristics, acid-producing rate, and acetic acid resistance of different strains were closely linked to PQQ-ADH. PQQ-ADH is a unique member of the alcohol dehydrogenase family and is essential for AAB oxidation of ethanol into acetic acid (Chen et al., 2016).

Previous studies of membrane-dependent ethanol oxidation, together with two-dimensional polyacrylamide gel electrophoresis (2D-PAGE) and mass spectrometry, have promoted the use of proteomic analysis to study acetic acid resistance by AAB (Trček et al., 2015; Wang et al., 2015). Proteomic analysis of *Acetobacter aceti* and *Gluconobacter oxydans* revealed that eight proteins were up-regulated under acetic acid stress conditions (Lasko et al., 1997). Additionally, at least 50 types of proteins were found to change significantly in response to acetic acid stress in a comprehensive protein expression analysis (Steiner and Sauer, 2001). In *A. aceti*, two proteins were seen to be significantly up-regulated by 2D-PAGE, one was aconitase and the other was a putative ABC transporter. Further investigations revealed that over-expression or absence of the genes encoding for these two proteins had an important influence on *A. aceti* acid resistance (Nakano and Fukaya, 2008). Fifty three relevant proteins were identified using 2D-PAGE for comparing the proteome of *A. pasteurianus* LMG1262T during acetic fermentation, growing *A. pasteurianus* in ethanol containing broth (Andrés-Barrao et al., 2012). These studies demonstrated the effectiveness of using 2D-PAGE to study the membrane-dependent pathway associated with acetic acid production.

The metabolism of acetic acid in *A. pasteurianus* mainly includes the Embden-Meyerhof-Parnas (EMP) pathway, pentose phosphate pathway (PPP), pyruvate metabolism pathway, ethanol oxidation respiratory chain pathway, and tricarboxylic acid cycle pathway (TCA) (Illeghems et al., 2013; Wang et al., 2015). The establishment of effective metabolic pathways is the basis of quantitative analysis of metabolic logistics and its regulation, which can provide a guarantee for the successful implementation of Metabolic Flux Analysis (MFA) (Dandekar et al., 2014).

Here, to investigate the effect of PQQ-ADH on acetic acid production, a strain that over-expressed PQQ-ADH subunits I (adhA) and II (adhB) was constructed, and the ethanol tolerance and acetic acid production of the engineered and original strains was compared. Furthermore, to find out differences in protein expression from the two strains, the type and function of the different proteins were inferred by comparison to mass spectrometry databases. And the MFA combined with proteomic analysis was performed to evaluate the relationship between the partial reformation of PQQ-ADH and whole metabolism of intrinsic cells, which was also the biggest innovation and highlights of this study.

## Materials and methods

### Materials

*Acetobacter pasteurianus* JST-S was previously identified in a screen performed in our laboratory from *A. pasteurianus* CICC 20001. 16S rDNA gene sequencing had been deposited in the GenBank database (accession number: MF457917). The plasmids used in this study are listed in Table 1 (Krahulec et al., 2003). All bacterial strains and plasmids were obtained from the Collection of the School of Food Science and Engineering, Hefei University of Technology, Hefei, China.

**Table 1:** Bacterial strains and plasmids used in this study.

**Strains or plasmids**	**Relevant characteristics^a^**	**Source or references**
**STRAIN**		
*Escherichia coli* JM109	*recA1, endA1, gyrA*96, *thi, hsdR*17, *supE*44,*relA*1, λ^−^, Δ(*lac*-*proAB*), [F′, *traD36 proAB, lacI^*q*^*ZΔM15]	Kanchanarach et al., 2010
**PLASMIDS**		
pEASYTM-T1	Ap^r^, Km^r^, f1 ori	TransGen Biotech^b^
PBBR1MCS-4^c^	Ap^r^, ColE1 ori PBBR1MCS-4 carrying Padh	Yanisch-Perron et al., 1985; Kovach et al., 1995
PBBR-adhA	PBBR1MCS-4 carrying adhA	This study
PBBR-adhB	PBBR1MCS-4 carrying adhB	This study
PBBR-adhA-adhB	PBBR1MCS-4 carrying adhA and adhB	This study

a *Ap^r^, ampicillin resistance; Km^r^, kanamycin resistance*.

b *TransGen Biotech, Beijing, China*.

c *A 367-bp fragment containing Padh, the adh promoter was amplified by PCR and cloned into PBBR1MCS-4, as described in Kovach et al. (1995) (Figure 1)*.

Seeds medium (YG_1_) contained 11 g L^−1^ of glucose, 11 g L^−1^ of yeast extract, 1.1 g L^−1^ of MgSO_4_·7H_2_O, 3.3 g L^−1^ of K_2_HPO_4_, and 2% (v v^−1^) ethanol. Fermentation medium (YG_2_) contained 5 g L^−1^ of glucose, 5 g L^−1^ of yeast extract, 1.1 g L^−1^ of MgSO_4_·7H_2_O, 3.3 g L^−1^ of K_2_HPO_4_, and different concentrations of ethanol. YPGD medium contained 5 g L^−1^ yeast extract, 5 g L^−1^ peptone, 5 g L^−1^ glucose, 5 g L^−1^ glycerol, 17 g L^−1^ agar, and 3.5% (v v^−1^) ethanol. Media were divided into 50 ml and poured into 250-ml Erlenmeyer flask. Then, they were sterilized at 121°C for 20 min prior to the addition of ethanol. Cells were seeded in YG_1_ and cultured on intelligent thermostatic shaking incubator (ZHP-Y2102L, Shanghai Sanfa Scientific Instruments Co. Ltd., Shanghai, China) at 30°C for 36 h at 170 rpm. Fermentation cultivations were performed in YG_2_ medium at 32°C at 170 rpm.

Restriction enzymes, DNA ligases, low melting-point agarose, DNA gel extraction kit, polymerase chain reaction (PCR) purification kit, and other genetic engineering-related reagents were purchased from TaKaRa (TaKaRa Biotechnology Co., Ltd., Dalian, China). 2D-PAGE-related reagents, protein standards, and 17-cm (pH 4-7) IPG pre-prepared adhesive strips were obtained from Bio-Rad (Bio-Rad Laboratories, Inc., Hercules, USA). Chemicals used in this study were provided by Songong Biotech (Shanghai, China) Co., Ltd.

### Construction of PQQ-ADH over-expression strain

*E. coli* plasmids were extracted with a MiniBEST Bacterial Genomic DNA extraction kit Ver.2.0 (TaKaRa Biotechnology Co., Ltd., Dalian, China) and stored at −20°C. A Mini BEST Plasmid Purification Kit Ver.4.0 (TaKaRa Biotechnology Co., Ltd., Dalian, China) was used for the preparation and transformation of *E. coli* competent cells. Standard methods were used for *A. pasteurianus* JST-S genomic DNA extraction, cell collection, and cell lysis. For amplification of Padh (the promoter region of the operon coding for subunits I and II of PQQ-ADH from *A. pasteurianus*), adhA and adhB, primer sequences were designed using Primer Premier 5.0 software (Premier Biosoft, CA) (Table 2). PCR and vector construction methods are detailed elsewhere (Matsushita et al., 1992; Krahulec et al., 2003; Andrés-Barrao et al., 2012; Zheng et al., 2014). The construction strategy and sketch map of recombinant plasmids are shown in Figure 1.

**Table 2:** Primer sequences.

**Primers**	**Sequence**	**Restriction site**
adhA-P1	CGCGGATCCATGACCCGCCCCGCCTCCGCCAAGA	BamH I
adhA-P2	TGCACTAGTTTAGGGGTTAATGCCAAGTGTCG	Spe I
adhB-P1	CGCACTAGTGATGATGATGAACAGGCTAAAAACTGC	Spe I
adhB-P2	AGCTCTAGATTACTGGGCTTCATCCACACCAGCA	Xba I
Padh-P1	CGCGGATCC ATCCACCACAGCCTGCGTGCACCAGA	BamH I
Padh-P2	AGCACTAGTGCGTTCATGTCCTCGACTATTATATA	Spe I

*The underlined sequences represent the restriction sites*.

**Figure 1:**
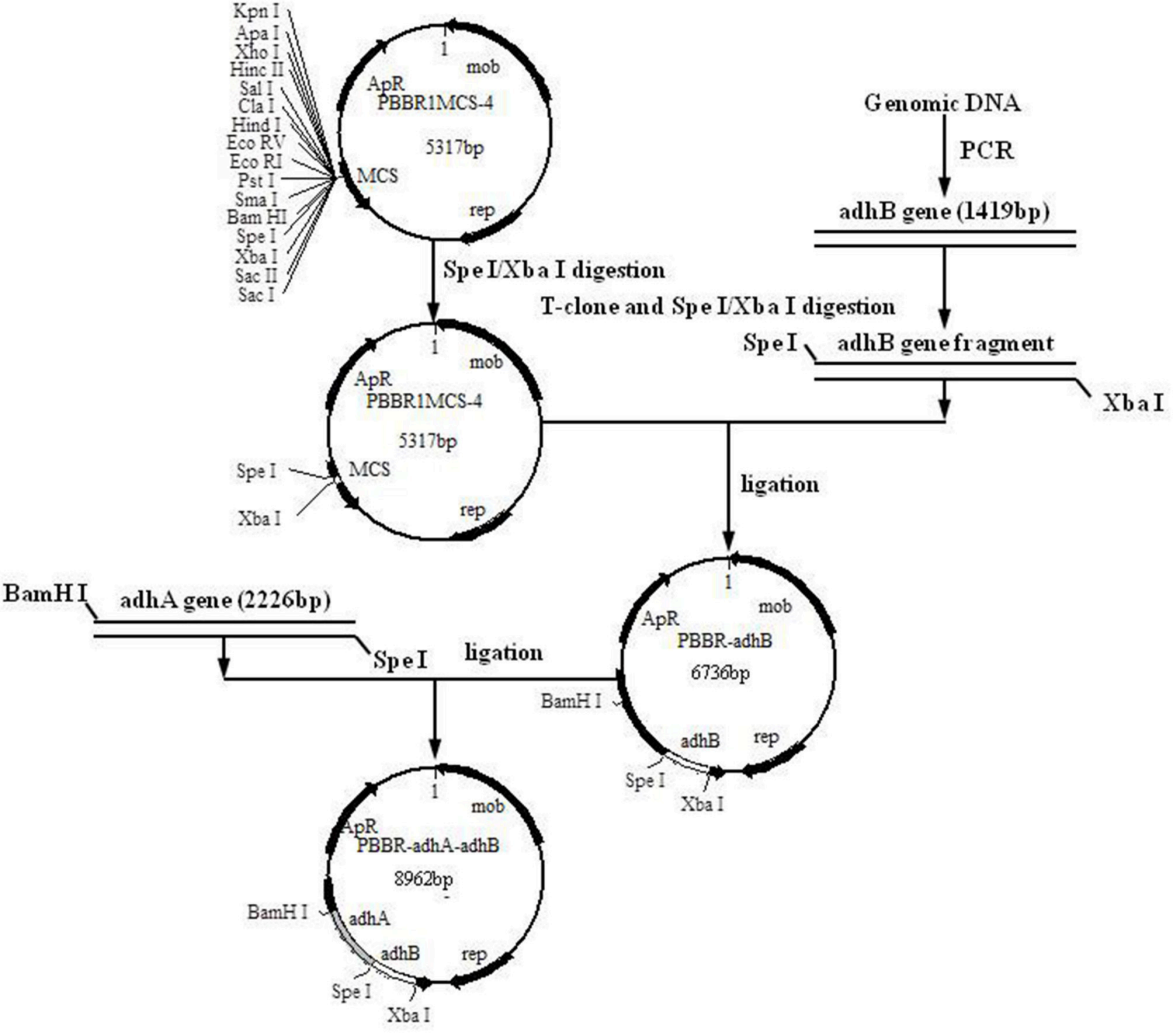
Construction of recombinant plasmid PBBR-adhA-adhB.

Competent *A. pasteurianus* JST-S cells were prepared according to the references (Matsushita et al., 1992; Andrés-Barrao et al., 2012). Plasmids were introduced into *A. pasteurianus* JST-S by electroporation using a MicroPulser Electroporation Apparatus (Bio-Rad Laboratories, Inc., Hercules, USA) in an ice-water bath at 2.5 KV. After the shock, cells were quickly transferred to YG_1_ and cultured at 170 rpm for 2 h at 30°C. Subsequently, the samples were cultured in YPGD medium containing 100 μg ml^−1^ ampicillin, at 30°C for 48 h to select for strains which containing recombinant plasmid.

### Determination of enzymatic activity

Strains were incubated in YG_2_ with a gradient ranging initial ethanol (0, 1, 2, 3, 4, 5, 6, 7, 8%) (v v^−1^) at 32°C for 48 h at 170 rpm. Then, the fermentation broth was centrifuged by centrifuge (HC-3018R, Anhui USTC Zonkia Scientific Instruments Co., Ltd., Anhui, China) at 8,000 × g for 10 min at 4°C to collect cells. The samples were washed three times with 0.1 M phosphate buffer saline (PBS pH 7.0, 3 ml buffer per one gram of wet bacteria) with the same centrifugal condition and resuspended in the same buffer. Subsequently, the suspension was broken into some active fragments by JY92-II ultrasonic disintegration (220 W, 3s 3s^−1^) (Ningbo Scientz biotechnology Co., Ltd, Zhejiang, China) for 10 min in ice-water bath. Fractured fluid was further centrifuged at 10,000 × g for 30 min at 4°C to get supernatant fraction as enzyme liquid. The activity of ADH was measured as previously described (Toyama et al., 2004; Trček et al., 2006; Matsushita et al., 2008; Qi et al., 2014). One unit of ADH enzyme activity is defined as the amount of enzyme demanded to catalyze the oxidation of 1 u mol of substrate per minute. Determination of ADH enzyme was performed at 25°C. The protein concentration was measured by the modified Lowry method, with bovine serum albumin as a standard protein (Trček et al., 2006; Yakushi and Matsushita, 2010).

### Assays of total acid content and ethanol content

The fermentation cultivation was conducted in YG_2_ with 42 g L^−1^ ethanol content (EC) and 10 g L^−1^ acetic acid content, been shaked at 170 rpm at 32°C for 96 h. The acidity of fermentation broth was measured by 0.1 M NaOH with phenolphthalein as pH indicator (Chinnawirotpisan et al., 2003; Krusong et al., 2015). Ethanol content assays were performed by Agilent 7890A GC System (Agilent Technologies Investment Co., Ltd., Shanghai, China) with a 30 m × 1.25 mm × 0.4 μm DB624 capillary column. N-butyl alcohol (0.1 ml n-butyl alcohol per milliliter of fermentation broth) was added as an internal standard. The flow rate of carrier gas (nitrogen) was 30 ml min^−1^. Injector and detector temperatures were 200°C and 250°C, respectively. The temperature program of capillary column GC finally was set consisted of 100°C for 1 min, ramp to 190°C at 15°C min^−1^ and hold for 3 min. According to the internal standard curve and the peak area ratio of ethanol and n-butanol alcohol in the sample, ethanol content of fermentation broth was calculated.

### Determination of glucose and acetic acid

High performance liquid chromatography (HPLC) was performed for determining the concentration of glucose and acetic acid in the fermentation broth. The sample was centrifuged at 25°C for 10 min at 10,000 × g, and 1 ml of the supernatant was diluted with distil-water and filtered through a 0.22 μm membrane. An Atlantis dC_18_ 150 mm × 4.6 mm (3.0 um) chromatographic column (Waters, USA) was used as analytical column with a flow rate of 0.6 ml min^−1^, the column temperature was 30°C (Li et al., 2014; Qin et al., 2016; Zhang et al., 2017). 0.05 M NH_4_H_2_PO_4_ buffer adjusted to pH value 2.5 with phosphoric acid was conducted as mobile phase. Concentration of glucose was analyzed by HPLC Waters 600 (American Waters Co., Milford, USA) equipped with UV detector (Waters 2487) at 210 nm, and acetic acid production was measured by HPLC Waters 600 (American Waters Co., Milford, USA) equipped with refractive index detector (Waters 2414) at 40°C. The injection volume of samples was 10 μl.

### 2D-page analysis

Protein samples were extracted as indicated elsewhere (Nakano and Fukaya, 2008; Andrés-Barrao et al., 2012). The different steps of isoelectric focusing were performed as follows: 50 V hydration for 12 ~16 h, 250 V linear run for 30 min, 1,000 V fast run for 1 h, 8,000 V linear run for 5 h, and 8,000 V fast run until a total of 70,000 V h^−1^. The strips (17 cm, pH 4-7, Limiting current of each strip 30–50 μA) were incubated for 15 min in 10 ml equilibrium solution (0.375 M Tris-HCl pH 8.8, 6 M urea, 2% (w v^−1^) SDS, 20% (v v^−1^) glycerol), +2% (w v^−1^) DDT then, in equilibrium solution +2.5% (w v^−1^) iodoacetamide. SDS-PAGE was performed in a Protean II electrophoresis tank for 6–8 h at 30 m Agel^−1^, until the front of the bromophenol blue marker reached the bottom of the glass. After 2D-PAGE, the gel was stained immediately with Coomassie brilliant blue R-250 for 40 min at 25°C on a horizontal shaker, followed by destaining (three times, 30 min each). Once destaining was complete, 2D-PAGE maps were scanned and analyzed using a 2100XL-USB scanner (UMAX, Taiwan) and PDQuest 8.0 software (Bio-Rad, USA), respectively. Differentially expressed spots were sent to the Life Science Laboratory Center of the University of Science and Technology of China for mass spectrometry analysis.

### Metabolic flux analysis

The suggested metabolic network was constructed by using the substrate utilization and combining our results on enzyme protein expression and genomic information from the Kyoto Encyclopedia of Genes and Genomes (http://www.kegg.jp) (Li et al., 2009; Antoniewicz, 2015; Wallenius et al., 2016). Metabolic flux (MF) analysis, which is based on the pseudo-steady-state assumption that there is no accumulation of any intermediates for a certain period of time, was performed for the calculation of volumetric rates of intracellular metabolite formation (Stephanopoulos, 1999). The total stoichiometric matrix of all reactants and reaction products (A) was calculated as follows:


(1) $$\begin{array}{r} {\text{A}}^{\text{T}}\text{B}=\text{R} \end{array}$$

A represented the total stoichiometric matrix for all reactangts and products of reactions. B stood for the internal reaction rates (mmol g_DW_^™1^h^−1^), and R stood for the net formation rate of metabolites (mmol g_DW_^™1^h^−1^). MF was computed using MINVERSE and MMULT in Excel 2010 (Microsoft Co., Redmond, WA, USA) (Li et al., 2009).

## Results and discussion

### Over-expression of PQQ-ADH in *A. pasteurianus* JST-S

#### Cloning and analysis of padh, adhA, and adhB sequences

**Figure 2:**
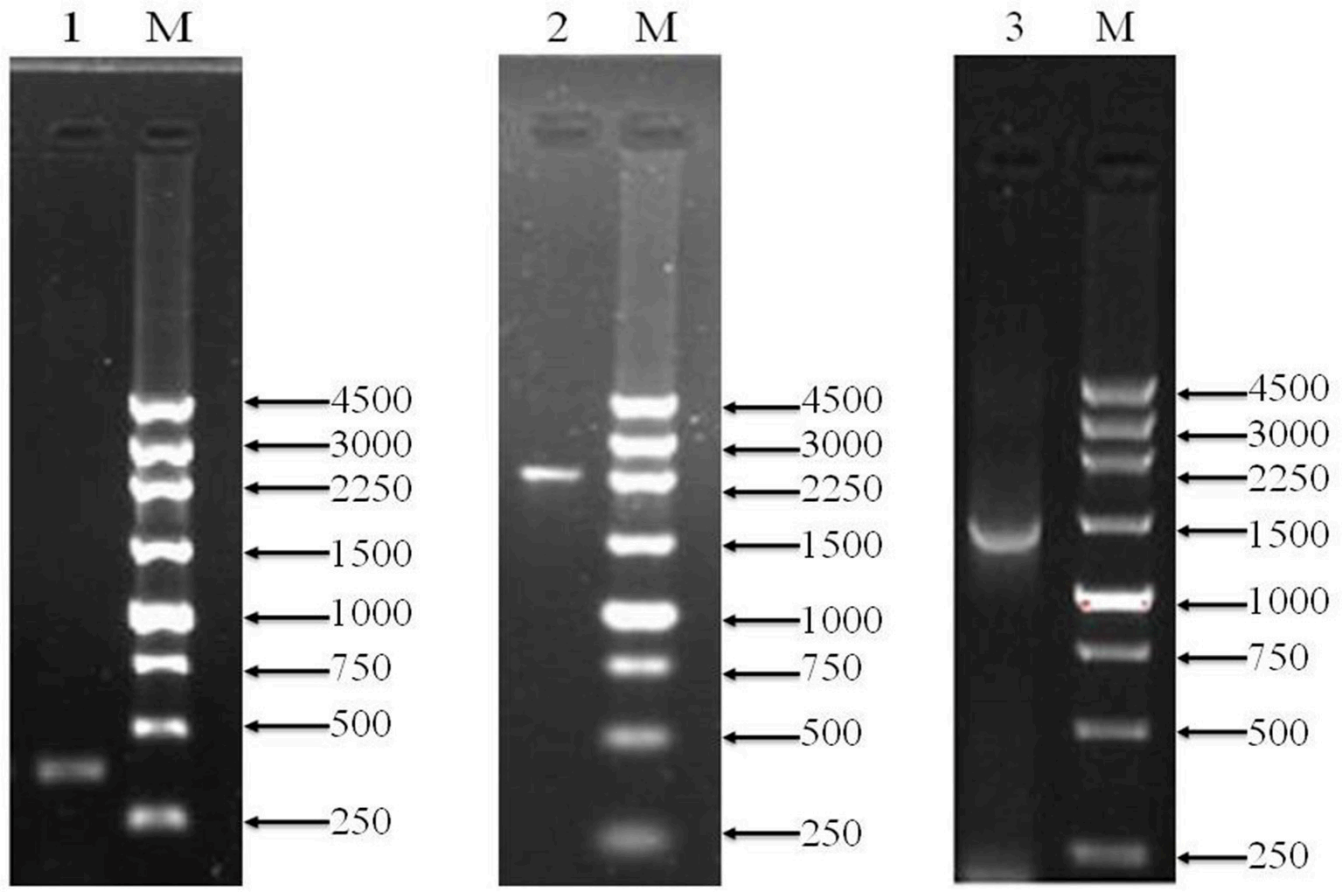
PCR amplification products shown by agarose gel electrophoresis. Lanes M, marker (bp);1, Padh; 2, adhA; 3, adhB.

To over-express PQQ-ADH, we subcloned Padh, adhA, and adhB into a high-expression vector. Previous work had shown that Padh was essential for the expression of adhA and adhB, as its absence prevented acetic acid production. Accordingly, Padh (Zheng et al., 2014) was amplified from *A. pasteurianus* JST-S and cloned into PBBR1MCS-4 (5317 bp) as shown in Figure 1. Padh, adhA, and adhB were amplified from the genomic DNA of *A. pasteurianus* JST-S by PCR using previously published primers (Zheng et al., 2014). The PCR products were analyzed by electrophoresis. As shown in Figure 2, Padh, adhA, and adhB were about 367, 2,226, and 1,419 bp in length, respectively. Sequence analysis showed that the adhA fragment contained only a single reading frame (ORF) of 2,226 bp, with an ATG start codon and a TAA stop codon. We reasoned that adhA and adhB ORFs encoded 742 and 473 amino acid residues, respectively. The cloned adhA (GenBank database ID: 1992895 Seq1 KY643658) sequence had 98.7% of identity to the *A. pasteurianus* adhA sequence deposited in GenBank (GenBank database ID: 16503088) (Zheng et al., 2014), that indicated the cloned fragment was indeed the adhA fragment of *A. pasteurianus*. The same result of adhB (GenBank database ID: 1983884 Seq1 KY584296).

### Construction and identification of recombinant plasmids

**Figure 3:**
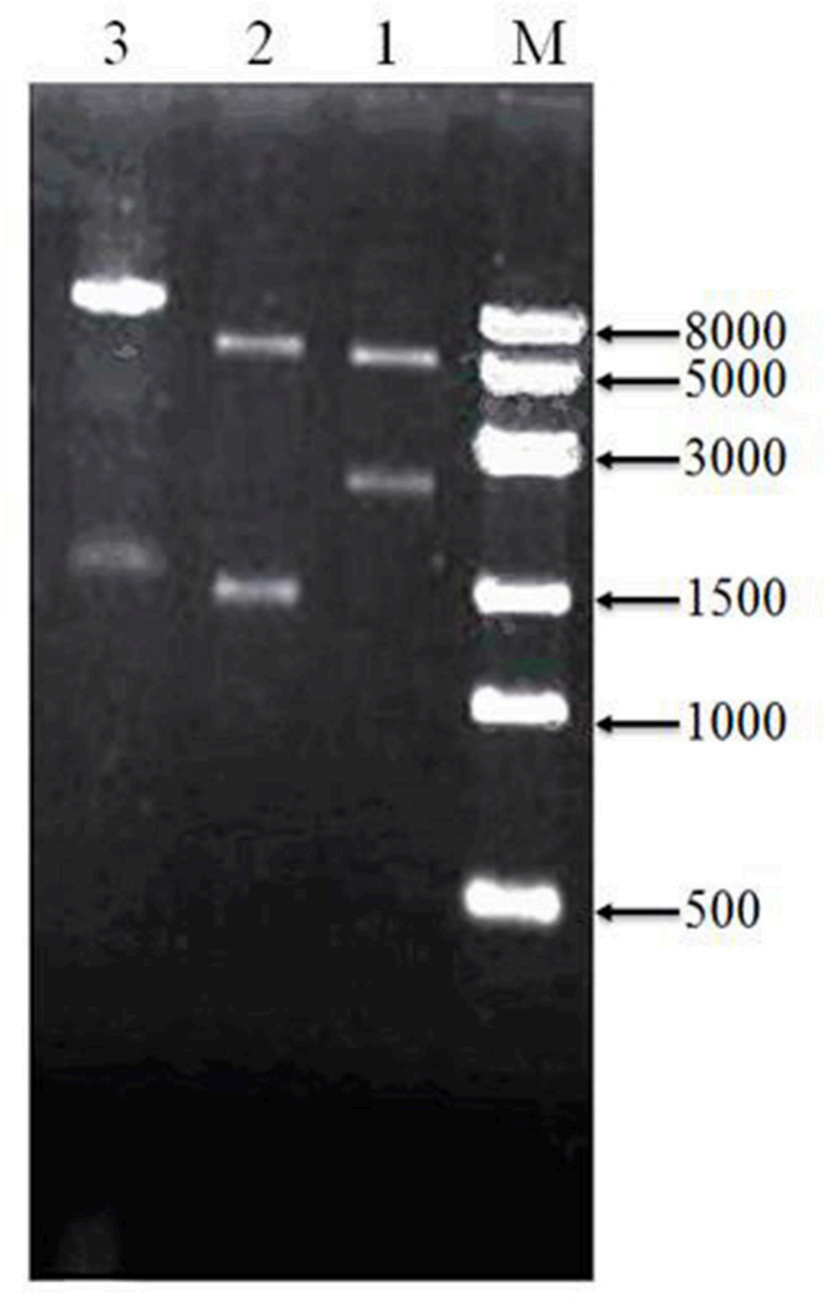
Verification of recombinant plasmids by restriction enzyme digeston. Lanes: M, 8-kbp marker, 1, PBBR-adhA digested with Bam HI and Spe I; 2, PBBR-adhB digested with Spe I and Xba I; and 3, PBBR-adhA-adhB digested with Bam H I.

To assess cloning accuracy, the recombinant plasmids were verified by double digestion with restriction enzymes (Figure 3). As seen in Lane1, PBBR-adhA produced a band compatible with the expected 2,226-bp fragment. Digestion of PBBR-adhB (Lane 2) produced a band that also matched the expected size (1,419 bp). Finally, PBBR-adhA-adhB digestion with BamH I (Lane 3) generated a large fragment similar to the expected linear plasmid (8,962 bp). In summary, digestion patterns showed that the recombinant plasmids were constructed successfully.

### Assessment of adhA-adhB over-expression

#### Verification of adhA-adhB over-expression

**Figure 4:**
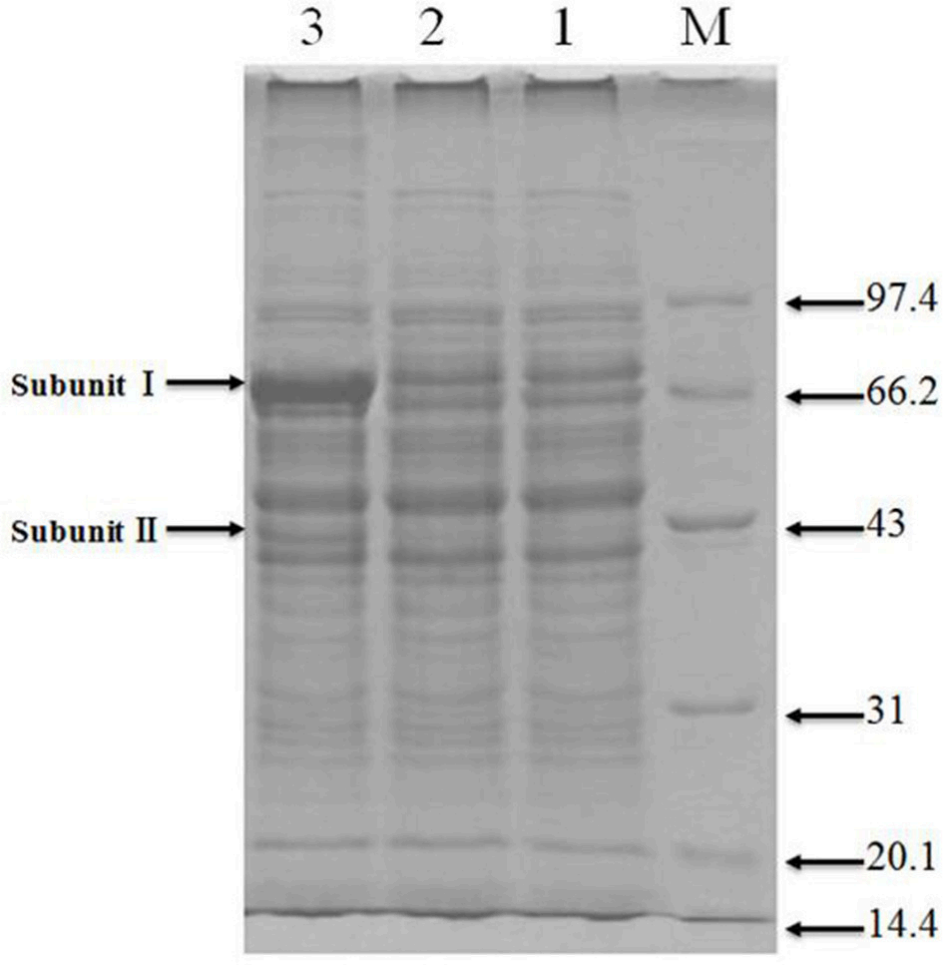
SDS-PAGE of recombinant protiens over-expressed in the genetically engineered strain Lanes: M, molecular weight marker (KDa); 1 and 2, original strain; 3, engineered strain.

Total protein extracts from the original and engineered strains were separated by SDS-PAGE (Figure 4). As can be observed in Lane 3, the engineered strain displayed bands compatible with the size expected for ADH subunits I and II, indicating the successful expression of adhA and adhB.

#### Verification of ADH enzymatic activity in the engineered strain

**Figure 5:**
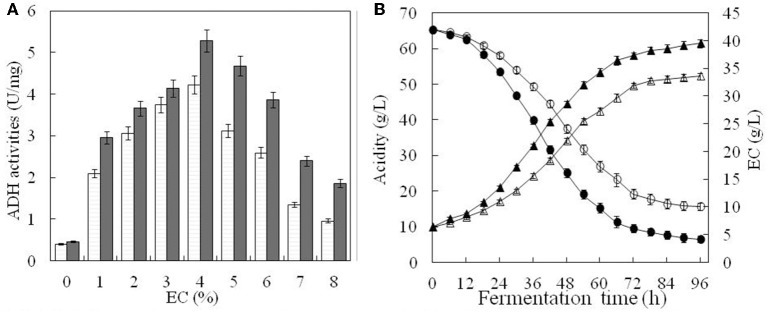
Comparison of fermentation parameters in the original and engineered strains. ADH activity of the original strain(□) and engineered strain(■) were compared under the different content of ethanol in **(A)**. In **(B)**, white triangle and black triangle represent total acid content of the original strain and engineered strain, respectively. Similarly, white circle and black circle represent ethanol content of the original strain and engineered strain, respectively. Error bars show standard deviations of three replicated measurements.

ADH activity changed under different EC as shown in Figure 5. In the absence of ethanol, ADH activity was 0.40 U mg^−1^ for the original strain and 0.46 U mg^−1^ for the engineered one (Figure 5A). When EC increased from 1 to 4%, ADH activity of both strains also increased. The highest activity was observed at 4% of EC, corresponding to 4.23 U mg^−1^ and 5.28 U mg^−1^, respectively, for the original and engineered strains (Figure 5A). For EC above 4%, ADH activity decreased rapidly in both strains, reaching its lowest level at 8% of EC. At this point, ADH activity of the engineered strain (1.86 U mg^−1^) was ~1.9 times that of the original strain (0.96 U mg^−1^) (Figure 5A). In general, ADH activity of the engineered strain was higher than that of the original strain at any given EC, suggesting that ADH expression was also increased.

### Total acid content in the original and engineered strains

Total acid content and ethanol consumption in both strains were relatively low during the first 12 h of cultivation, probably due to *A. pasteurianus* having adapted to a high concentration of ethanol (Figure 5B). Between 12 and 72 h, acetic acid accumulated rapidly, while EC decreased with a steady rate. After 72 h, nearing the end of fermentation, accumulation of acetic acid slowed and EC remained almost unchanged. For the engineered strain, the final total acid content was 61.42 g L^−1^ and EC was 4.18 g L^−1^. For the original strain, total acid content was only 52.23 g L^−1^ and EC was 10.11 g L^−1^. This indicated that total acid content and ethanol conversion were higher in the engineered than the original strain under the same conditions. It also suggested that ADH activity was enhanced in the engineered strain.

### Proteomic analysis of differentially expressed proteins

#### Proteomic analysis

**Figure 6:**
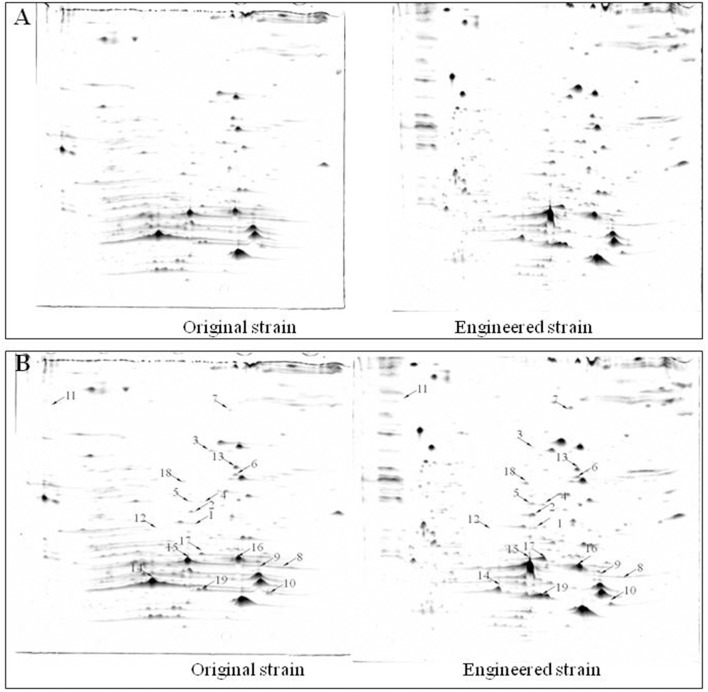
Comparison of 2D-PAGE patterns between original engineered strains. **(A)** Primary 2D-PAGE maps. **(B)** 2D-PAGE maps analyzed using PD Quest 8.0 software. Protein spots whose expression level changed more than 50% were identified as differentially expressed proteins.

Protein extracts from the original and engineered strains cultivated in YG_2_ at 170 rpm at 32°C for 48 h were analyzed by 2D-PAGE (IPG 4-7, 17-cm strips followed by SDS-PAGE) (Figure 6). Nineteen representative differentially expressed protein spots were identified.

#### Functional analysis of differentially expressed proteins

Differentially expressed protein spots were analyzed by tandem mass spectrometry (MS/MS) and the peptide fingerprint spectrum was identified. The Swiss-Prot protein database (http://www.uniprot.org/) was used to search for peptides; 17 of the 19 queried peptides matched those in the database.

Functional analysis of identified protein spots confirmed significantly higher levels of PQQ-ADH (adhA and adhB) in the engineered compared to the original strain. This was consistent with SDS-PAGE analysis (Figure 4). Overall, we identified 13 up-regulated and four down-regulated protein spots; two additional spots could not be identified (Table 3). PQQ-ADH, chaperonin, and acetaldehyde dehydrogenase were particularly up-regulated, indicating that PQQ-ADH over-expression enhanced the ethanol oxidation respiratory chain (Illeghems et al., 2013).

**Table 3:** Protein identification using MS/MS peptide fragmentation and Swiss-Prot database search.

**Spot^a^**	**Swiss-prot accession number**	**Identified protein^b^**	**Fold change^c^**	**Length**	**Mass (kDa)^d^**	**pI^e^**	**Mascot score^f^**	**Functional classification**
1	C7JC25	Heat shock protein DnaK	2.05	634	67.5	4.9	1965	coping with heat or external environmental pressure
2	C7JBN6	Trigger factor	4.52	444	49.3	4.78	580	peptidyl-prolyl cis-trans isomerase
3	C7JAP1	Thioredoxin	2.57	319	34.4	4.88	317	protein disulfide oxidoreductase activity
4	C7JDY6	Glutamine synthetase	3.64	481	53.1	5.34	147	ATP binding
5	C7JAQ5	Aldehyde dehydrogenase	6.32	463	50.6	5.27	1061	oxidoreductase activity
6	Q5HXK5	NAD(P)H-dependent 2-cyclohexen-1-one reductase	−2.79	354	39.3	5.96	1056	oxidoreductase activity
7	C7JBD3	Alkyl hydroperoxide reductase AhpD	5.73	175	18.4	5.87	576	response to oxidative stress
8	C7JAW8	Alcohol dehydrogenase large subunit	5.05	742	81.6	6.77	305	oxidoreductase activity
9	C7JG48	Aconitate hydratase	−2.34	897	97.7	6.21	1098	aconitate hydratase activity
10	C7JFQ0	50S ribosomal protein L1	4.17	230	23.9	6.87	702	direct binding to 23S rRNA
11	NI	Uncharacterized protein	NI	NI	NI	NI	NI	NI
12	C7JG40	Transcription termination/antitermination protein NusA	−2.46	514	57.6	4.61	348	translation elongation factor activity
13	C7JIQ0	NADH:flavin oxidoreductase	−3.12	357	39.5	5.96	557	oxidoreductase activity
14	F1YU43	Chaperonin	5.32	546	58	5.49	576	ATP binding
15	M9WQJ0	Elongation factor Tu	2.69	836	74.9	5.22	158	translation elongation factor activity
16	C7JCS6	Thiosulfate sulfurtransferase	3.67	292	32.5	5.54	79	transferase activity
17	A0A0K0TA70	S-adenosylmethionine synthase	2.73	960	68.5	5.3	154	ATP+L-methionine+H_2_O = phosphate+diphosphate+S-adenosyl-L-methionine
18	C7JDV2	Esterase/lipase	3.12	388	40.8	4.98	254	lipolysis
19	NI	Uncharacterized protein	NI	NI	NI	NI	NI	NI

a *Number as shown in Figure 6*.

b *Protein spots whose expression level changed more than 50% as calculated by PDQuest 8.0 software. Nineteen spots were identified, of which 17 were researched and named as described in Table 3*.

c *Fold changes in the expression of proteins between original and engineered strains. A minus sign indicates down-regulation, otherwise it denotes up-regulation*.

d *Theoretical protein molecular mass as derived from amino acid sequences*.

e *Isoelectric point of the protein as calculated from amino acid sequences*.

f *Percentage of predicted protein sequence covered by matched peptides via MASCOT (http://www.matrixscience.com/)*.

*NI represented the protein spot could not be identified*.

Spot 1 in Table 3 was identified as a DnaK heat shock protein. DnaK plays an important role in preventing protein denaturation, and restoring the original structure and biological activity, especially under heat shock or other stressful conditions. Heat shock protein up-regulation has been shown to coincide with resistance to external stresses, such as high acetic acid or other harmful environmental factors (Welch, 1993; Hartl and Hayer-Hartl, 2002). The behavior of AAB heat-shock proteins, such as GroES, GroEL, DnaK, and DnaJ, in response to changes in temperature, alcohol content, acidity and prevent protein misfolding have been studied before (Okamoto-Kainuma et al., 2002, 2004). Accordingly, the increased ethanol tolerance of the engineered strain may be explained by the up-regulation of heat shock proteins.

Spot 2 corresponded to a trigger factor with peptidyl-prolyl cis-trans isomerase activity. This factor can interact with short peptide chains by binding to the ribosome and thus facilitate the correct folding of newly synthesized peptides (Hesterkamp et al., 1996; Hartl and Hayer-Hartl, 2002).

Spot 4 corresponded to glutamine synthetase. This enzyme catalyzes the conversion of ammonium and glutamic acid to glutamine, which is involved in protein synthesis and transport of ammonia. The catalytic reaction of glutamine synthetase can be divided in two steps. In the first, ATP reacts with glutamic acid to form γ-glutamic acid. In the second, the phosphate group is replaced by the ammonium ion to yield glutamine (Rhee and Chock, 1976). Recently, glutamine has been shown to control intracellular pH by neutralizing protons under highly acidic conditions in *E. coli*. The underlying mechanism depends on its conversion into glutamate and the release of ammonia (Lu et al., 2013). The up-regulation of glutamine synthetase may play an important role in preventing intracellular acidification under elevated acid stress.

We observed a down-regulation of oxidoreductases, such as NAD(P)H-dependent 2-cyclohexen-1-one reductase (spot 6) and NADH: flavin oxidoreductase (spot 13). These are known to be involved in bacterial growth; their down-regulation may indicate that ADH over-expression and increased acid production decrease cell growth. Some studies have suggested that the physiological effect induced by changes in environmental stress factors, such as temperature, salt ions, and acidity, is mediated by the oxidoreductase pathway (Richier et al., 2006; Ryter et al., 2007). Furthermore, increased acidity in the environment causes a metabolic imbalance, those results in the production of a large amount of oxygen free radicals, which cause oxidative damage in the cells. Consequently, oxidoreductase activity could be used as an indicator of an organism's response to environmental stress. The up-regulation of thioredoxin (spot 3) responsible for maintaining disulfide bonds, and the corresponding metabolic implications will require further research.

Spot 7 corresponded to alkyl hydroperoxide reductase, a member of the peroxiredoxin family responsible for the detoxification of active substances. To protect the cell and reduce peroxides, alkyl hydroperoxide reductase uses mainly the electrons donated by NADPH and the FAD reductase AhpF.

Aconitase (spot 9) is a coenzyme that catalyzes the conversion of citric acid to isocitrate and participates mainly in the tricarboxylic acid cycle (Nakano and Fukaya, 2008; Andrés-Barrao et al., 2012). Down-regulation of aconitase suggested reduced tricarboxylic acid cycle activity, possibly explaining why at the beginning of fermentation the engineered strain grew more slowly than the parent one. It has been suggested that improving aconitase expression could effectively increase acid producing by AAB (Nakano et al., 2004), however we found that PQQ-ADH over-expression actually down-regulated aconitase. Therefore, the link between aconitase levels and acid production in AAB will require further investigation.

Spot 15 was identified as a translation elongation factor Tu (EF-Tu) showed an up-regulated expression level, as shown in Table 3. It has a conserved structure spanning 836 amino acid residues. EF-Tu, an essential factor for protein synthesis promoted the GTP-dependent translocation of the nascent protein chain from the A-site to the P-site of the ribosome (Lariviere et al., 2001). This differential expression was associated with the significant increase in protein translation by constructing a engineered strain that over-expressed PQQ-ADH subunits I (adhA) and II (adhB).

Proteomic analysis revealed that aldehyde dehydrogenase (ALDH) (spot 5), the large subunit of ethanol dehydrogenase (spot 8), and a chaperonin (spot 14) were up-regulated. This was likely due to PQQ-ADH over-expression and increased flux through the ethanol oxidation respiratory chain pathway. Heat shock protein (spot 1), trigger factor (spot 2), glutamine synthetase (spot 4), alkyl hydroperoxide reductase (spot 7), and elongation factor Tu (spot 15) were also up-regulated in the engineered strain, possibly explaining its improved tolerance of environment stresses, such as acid, heat, and alcohol. However, aconitase, a key enzyme of the tricarboxylic acid cycle, was down-regulated, which could lead to lower tricarboxylic acid cycle activity and lower growth of AAB.

### Metabolic flux analysis

**Figure 7:**
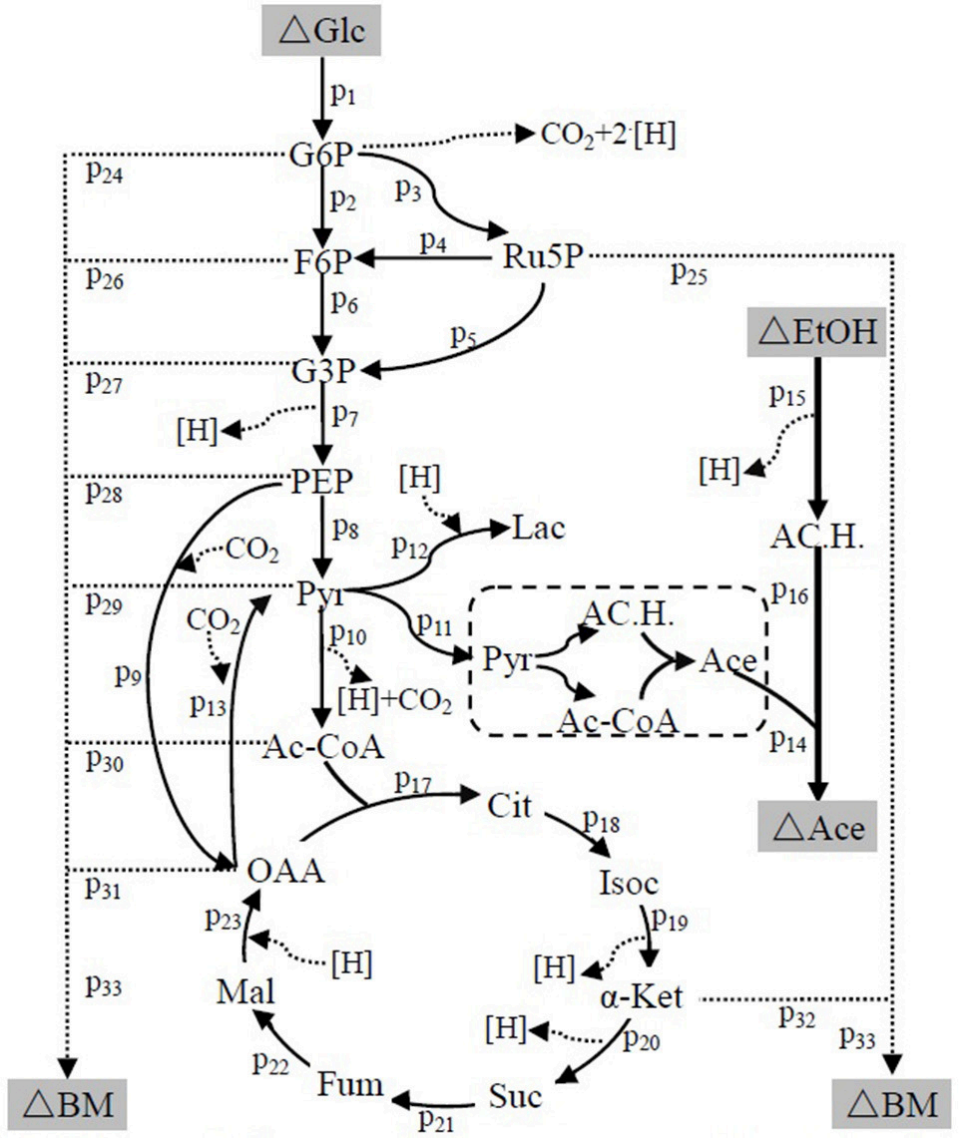
The metabolic network of *A. pasteurianus* JST-S Glc, glucose; G6P, glucose-6-phhospahte; F6P, fructose-6-phosphate; Ru5P, ribulose 5-posphate; G3P, glyceraldehydes-3-phosphate; PEP, poshonolpyruvate; Pyr, pyruvate; Pyr, pyruvate; AC.H., Acetaldehyde; OAA, oxaloacetate; Acetyl-CoA, acetyl coenzyme A; CoA, Coenzyme A; Lac, Lactate; Ace, Acetate; EtOH, ethanol; Cit, citrate; Isoc, Isocitrate; α-Ket, α-ketoglutaric acid; Suc, succinic acid; Fum, fumarate; Mal, Malate; BM, biomass.

The suggested metabolic network of *A. pasteurianus* JST-S with ethanol and glucose was shown in Figure 7 (Illeghems et al., 2013). All the biochemical reactions and enzymes are presented in Table 4, metabolic flux equations of *A. pasteurianus* JST-S corresponding to these reactions are shown in Table 5. A total of 33 equations were obtained from those biochemical reactions, following the basal mass balance. The coefficient of nine equations was obtained from previous studies (Longacre et al., 1997; Li et al., 2014), whereas that of all others was computed by dividing the net change in the concentration of metabolite during a short period of the nearly stationary phase by the duration of this phase. At 48–60 h, the fermentation rate was very high (As shown in Figure 5B), but the synthesis of cell biomass was almost stationary (data not show). Therefore, we selected the fermentation time of original strain and engineered strain for 48 h for the analysis of fluxes and identified a total of 33 fluxes, a number that was equal to that of biochemical reactions.

**Table 4:** Reactions and enzymes in the metabolic pathway of Acetic acid in *A. pasteurianus* JST-S.

**NO**.	**Enzyme**	**Metabolic reactions**
1	Glucokinase	ATP + Glc = ADP + G6P
2	Glucose-phosphate-isomerase	G6P = F6P
3	Glucose-6-phosphate dehydrogenase	G6P + 2 × NADP^+^ = 2 × NADPH + Ru5P + CO_2_
4	Transketolase/Transaldolase	3 × Ru5P = 2 × F6P + G3P
5	Phosphofructokinase /Aldolase/ Triose-phosphate-isomerase	ATP + F6P = ADP + 2 × G3P
6	Enolase /Phosphoglycerate-kinase / Phosphoglyceromutase /Glyceraldehydes -phosphate-dehydrogenase	G3P + NAD^+^ + Pi + ADP = PEP + NADH + ATP + H_2_O + H^+^
7	Pyruvate-kinase	ADP + PEP = ATP + Pyr
8	Phosphoenolpyruvate-carboxykinase	CO_2_+ PEP + ADP = OAA + ATP
9	Pyruvate dehydrogenase	Pyr + CoA + NAD^+^ = Ac-CoA + CO_2_+ NADH
10	Pyruvate decarboxylase	Pyr = AC.H. + CO_2_
11	Lactate dehydrogenase	Pyr + NADH + H^+^ = Lac + NAD^+^
12	Pyruvate carboxylase	ATP + Pyr + ${\text{HCO}}_{3}^{-}$ = ADP + Pi + OAA
13	Phosphotransacetylase /acetate kinase	Ac-CoA + Pi + ADP = CoA + Ace + ATP
14	Alcohol dehydrogenase	EtOH + NAD^+^ = AC.H. + NADH + H^+^
15	Acetaldehyde dehydrogenase	AC.H. + NAD^+^ = Ace + NADH + H^+^
16	Citrate synthase	Ac-CoA + H_2_O + OAA = Cit + CoA
17	Aconitase	Cit = Isoc
18	Isocitrate dehydrogenase	Isoc + NADP^+^ = α-Ket + NADPH + CO_2_
19	Oxoglutarate dehydrogenase complex	α-Ket + NAD^+^ = Suc + NADH + H^+^ + CO_2_
20	Fumarate reductase	Suc + FAD^+^ = Fum + FADH
21	Fumarate hydratase	Fum + H_2_O = Mal
22	Malate dehydrogenase	Mal + NAD^+^ = OAA + NADH + H^+^

*Glc, glucose; G6P, glucose-6-phosphate; F6P, fructose-6-phosphate; Ru5P, ribulose 5-phosphate; G3P, glyceraldehydes-3-phosphate; PEP, phosphoenolpyruvate; Pyr, pyruvate; AC.H., Acetaldehyde; OAA, oxaloacetate; Acetyl-CoA, acetyl coenzyme A; CoA, Coenzyme A; Lac, Lactate; Ace, Acetate; EtOH, ethanol; Cit, citrate; Isoc, Isocitrate; α-Ket, α-ketoglutaric acid; Suc, succinic acid; Fum, fumarate; Mal, Malate*.

**Table 5:** Metabolic flux equations of *A. pasteurianus* JST-S.

**Metabolic notes**	**NO**.	**Flux equations**	**Metabolic notes**	**NO**.	**Flux equations**
G6P	1	p_1_−p_2_−p_3_−p_24_ = 0	Fum	18	p_21_−p_22_ = 0
	2	p_2_−p_3_ = 0	Mal	19	p_22_−p_23_ = 0
F6P	3	p_2_+p_4_−p_6_−p_26_ = 0	OAA	20	p_9_−p_13_−p_17_+p_23_−p_31_ = 0
Ru5P	4	p_3_−p_4_−p_5_−p_25_ = 0	Biomass_G6P_	21	p_24_−0.005349p_33_ = 0
	5	p_4_−0.5 × p_5_ = 0	Biomass_F6P_	22	p_25_−0.001644p_33_ = 0
G3P	6	p_5_+p_6_−0.5 × p_7_−p_27_ = 0	Biomass_Ru5P_	23	p_26_−0.008948p_33_ = 0
PEP	7	p_7_−p_8_−p_9_−p_29_ = 0	Biomass_G3P_	24	p_27_−0.001291p_33_ = 0
	8	p_8_−6 × p_9_ = 0	Biomass_PEP_	25	p_28_−0.006889p_33_ = 0
Pyr	9	p_8_−p_10_−p_11_−p_12_+p_13_−p_29_ = 0	Biomass_Pyr_	26	p_29_−0.03604p_33_ = 0
	10	4 × p_12_−p_13_ = 0	Biomass_Ac−CoA_	27	p_30_−0.004139p_33_ = 0
Pyr → Ace	11	p_11_−p_14_ = 0	Biomass_OAA_	28	p_31_−0.01959p_33_ = 0
Ac-CoA	12	p_10_−p_17_−p_30_ = 0	Biomass_α−Ket_	29	p_32_−0.002744p_33_ = 0
AC.H.	13	p_15_−p_16_ = 0	Substrates	30	p_1_ = ΔGlc
Cit	14	p_17_−p_18_ = 0		31	p_15_ = ΔEtOH
Isoc	15	p_18_−p_19_ = 0	Products	32	p_14_+p_16_ = ΔAce
a-Ket	16	p_19_−p_20_−p_32_ = 0		33	p_33_ = ΔBM
Suc	17	p_20_−p_21_ = 0			

Δ*Glc, the glucose consumption;* Δ*EtOH, the ethanol consumption;* Δ*Ace, acetic acid production;* Δ*BM, the variation of biomass*.

Wet body weight of original strain and engineered strain was respectively 1.6 g L^−1^ and 1.54 g L^−1^ when fermentation time was 48 h at 32°C for 170 rpm. Original flux data of engineered strain and original strain was shown as in Table 6. The total flux of acetic acid production in engineered strain (8.2110 mmol g_DW_^™1^h^−1^) was higher than that of the original strain (5.2586 mmol g_DW_^™1^h^−1^), this was the result of the modification of ADH genes in *A. pasteurianus* JST-S. Thus, this further explained that the up-regulated expression of key enzymes in ethanol oxidation respiratory chain was in accord with the total flux data of acetic acid production. In Table 6, the flux of ethanol uptake in the original strain (5.1262 mmol g_DW_^™1^h^−1^) was lower that of engineered strain (7.8889 mmol g_DW_^™1^h^−1^), which meant the ethanol tolerance of engineered strain was better than that of original strain. That was what heat shock protein, trigger factor, glutamine synthetase, alkyl hydroperoxide reductase, and elongation factor Tu were up-regulated in the engineered strain (as shown in Figure 6, Table 3). The flux of glucose uptake in the original strain (0.2574 mmol g_DW_^™1^h^−1^) was higher than that of engineered strain (0.2337 mmol g_DW_^™1^h^−1^), indicating that the biomass of original strain was higher than that of engineered strain in the same fermentation time, this was consistent with the analysis of down-regulated oxidoreductases, such as NAD(P)H-dependent 2-cyclohexen-1-one reductase and flavin oxidoreductase (as shown in Figure 6, Table 3). The alcohol dehydrogenase gene of *A. pasteurianus* JST-S was modified by gene engineering might disturb the integrity of a bacterial gene sequence so that cell growth rate slowed down and the fluxes of various organic acids in TCA decreased comparing with the original strain (as shown in Table 6). The decrease in the fluxes of various organic acids in TCA might be due to the down-regulation of aconitase as a key enzyme of the tricarboxylic acid cycle (as shown in Figure 6, Table 3). These studies, combining proteome analysis with MFA, will make us easier to understand the effect of over-expressed PQQ-ADH on the metabolism of internal substances and may provide a reference for theory analysis of the metabolic mechanism with AAB.

**Table 6:** Original flux data of engineered strain and original strain.

**Flux data**	**Engineered strain**	**Original strain**	**Flux data**	**Engineered strain**	**Original strain**
p_1_	0.2337	0.2574	p_18_	0.0814	0.3169
p_2_	0.1146	0.1265	p_19_	0.0814	0.3169
p_3_	0.1146	0.1265	p_20_	0.0791	0.3146
p_4_	0.0357	0.0397	p_21_	0.0791	0.3146
p_5_	0.0714	0.0793	p_22_	0.0791	0.3146
p_6_	0.1489	0.1647	p_23_	0.0791	0.3146
p_7_	0.4384	0.4859	p_24_	0.0045	0.0045
p_8_	0.3708	0.4115	p_25_	0.0075	0.0075
p_9_	0.0618	0.0686	p_26_	0.0014	0.0014
p_10_	0.0849	0.3204	p_27_	0.0011	0.0011
p_11_	0.3221	0.1324	p_28_	0.0058	0.0058
p_12_	0.0145	0.0162	p_29_	0.0072	0.0072
p_13_	0.0578	0.0646	p_30_	0.0035	0.0035
p_14_	0.3221	0.1324	p_31_	0.0016	0.0016
p_15_	7.8889	5.1262	p_32_	0.0023	0.0023
p_16_	7.8889	5.1262	p_33_	0.8401	0.8401
p_17_	0.0814	0.3169			

*mmol g_DW_^−1^h^−1.^*

## Conclusions

To study PQQ-ADH over-expression, *A. pasteurianus* JST-S adhA and adhB were amplified by PCR, subcloned into a vector, and transformed back into *A. pasteurianus* JST-S. A comparison of the fermentation parameters indicated that acid production and ethanol conversion were higher in the engineered than in the original strain. The metabolic network was constructed and the MFA of the pathway from ethanol and glucose was also further investigated by comparing with original strain and engineered strain. Our study demonstrated that PQQ-ADH over-expression is an effective way to improve the acetic acid production in *A. pasteurianus*. Future experiments should aim at finding and analyzing the relative metabolic mechanism that are responsible for the tolerance of environment stresses.

## Author contributions

XW designed the study and wrote the protocol. HY conducted all experiments. MZ, XC, and JC took part in materials preparation. ZZ and LP took part in the manuscript preparation. LC and SJ performed the data analysis. ZW took part in polishing the manuscript. XL wrote and revised the manuscript.

### Conflict of interest statement

The authors declare that the research was conducted in the absence of any commercial or financial relationships that could be construed as a potential conflict of interest.
